# The effectiveness of HPV viral load, reflected by Cobas 4800 HPV-Ct values for the triage of HPV-positive women in primary cervical cancer screening: Direct endocervical samples

**DOI:** 10.1371/journal.pone.0232107

**Published:** 2020-05-07

**Authors:** Lyufang Duan, Hui Du, Chun Wang, Xia Huang, Xinfeng Qu, Bin Shi, Yan Liu, Wei Zhang, Xianzhi Duan, Lihui Wei, Jerome L. Belinson, Ruifang Wu

**Affiliations:** 1 Department of gynecology and obstetrics, Peking University Shenzhen Hospital, Shenzhen, China; 2 Shenzhen Key Laboratory on Technology for Early Diagnosis of Major Gynecological Diseases, Shenzhen, China; 3 Sanming Project of Medicine in Shenzhen Peking University Shenzhen Hospital, Shenzhen, China; 4 The Second Hospital of Hebei Medical University, Hebie, China; 5 Fudan University, Huashan Hospital, Shanghai, China; 6 Wuhan University, Zhongnan Hospital, Wuhan, China; 7 Capital Medical University Beijing Tongren Hospital, Beijing, China; 8 Peking University People’s Hospital, Beijing, China; 9 Preventive Oncology International, Cleveland Heights, Ohio, United States of America; 10 Women’s Health Institute, Cleveland Clinic, Cleveland, Ohio, United States of America; Rudjer Boskovic Institute, CROATIA

## Abstract

**Objective:**

To explore the relationship between the viral load reflected by the Ct value of Cobas 4800 HPV test and cervical lesions, and the effectiveness of the viral load for secondary triage of HPV-positive women.

**Methods:**

The Chinese Multi-Center Screening Trial (CHIMUST) evaluated both self-collected samples and physician-collected samples from women, aged 30 to 59, who were screened for cervical cancer in 6 regions across China. Using physician collected samples, the relationship between the HPV-Ct values of different subtypes and the cervical lesions was analyzed. Then the combined use of the HPV-Ct values with the HPV subtypes was evaluated as a secondary screening algorithm for the women who were HPV positive.

**Results:**

The Ct values of HPV16 and 12 other HPV subtypes(12-type pool), tested with Cobas decreased with the progression of cervical lesion (HPV16: *r* = -0.429, *P*<0.001; 12 other HR-HPV subtypes: *r* = -0.099, *P*<0.01). The HPV18-Ct value was not correlated with cervical lesion(*P*>0.05). Compared with HPV16/18 and cytology (HPV16/18 positive and 12-type pool plus cytology ≥ ASC-US), the sequential secondary screening using HPV16/18 and the viral load of 12-type pool (cut-point HPV-Ct≤31) had equal sensitivities for CIN2+ and CIN3+ (83.1%vs.80.3%,100%vs.92.6%,*P*>0.05), with slightly lower specificities (96.2%vs.94.4%,96.5%vs.93.9%,*P*<0.001) and higher colposcopy referral rate (4.90%vs.6.59%, P<0.05), but required no cytology.

**Conclusion:**

Type-specific HPV viral load is closely related to cervical lesions severity. It is feasible and efficient to use HPV16/18 and the viral load of 12 other HPV subtypes (with cut-point HPV-Ct≤31) as the secondary screening for HPV positive women. This algorithm may be useful in low resource regions.

## Introduction

Type specific persistence of high-risk human papillomavirus (HR-HPV) infection is the main cause of cervical cancer [1]. Therefore, HR-HPV testing has been widely used for cervical cancer screening. In recent years, HR-HPV testing as a primary cervical cancer screening method has been recommended [2]. At present, there is ample evidence that molecular biological testing of HR-HPV is more sensitive than cytology in the diagnosis of precancerous cervical lesions (CIN2/3 and AIS) or cervical cancer, and has a higher negative predictive value [3–6]. However, since most HPV infections are transient and can be cleared automatically, an unavoidable problem with HPV testing as a primary screening tool is its relatively low specificity and positive predictive value for high-grade cervical lesions [7,8]. This can easily lead to unnecessary colposcopy referrals and/or overdiagnosis and overtreatment. Therefore, a secondary screening methodology can serve a critical role for the HPV positive patients. A secondary triage test must be highly correlated with the progress or grade of cervical lesions in order to effectively stratify the risk of the disease. The correlation between high-grade cytological abnormalities, tumorigenic subtypes of HPV, and high-grade cervical lesions has been confirmed by many studies; and screening programs have been designed and developed around them [9,10]. For example the dual-staining of p16/ki67 in cervical exfoliated cell samples is highly predictive of high-grade cervical lesions [11,12]. However, the relationship between the HR-HPV viral load and the progression towards cervical cancer remains controversial. Several studies conducted by our team have shown that the higher the HR-HPV viral load, the higher the risk for high-grade cervical lesions [13,14] Recently, some authors have proposed secondary triage using viral load or combining viral load and genotyping for patients who test HPV positive in primary screening [14–17]. On review, most of the HPV testing technologies used in these studies were semi-quantitative assays, which do not distinguish high-risk HPV types. This makes it impossible to determine both the viral type and the viral load.

The Cobas^®^ 4800 (HR) HPV (hereinafter referred to as Cobas, Roche, Pleasanton CA) is the first HPV testing technology approved by the US FDA for primary cervical cancer screening. It specifically reports the presence of types 16 and 18, and then detects 12 other high-risk types as a pooled sample. Although Cobas is a qualitative testing technology for HPV, it can provide the cycle threshold (Ct value) of the virus copy number; that is, the number of cycles experienced by the fluorescent signal in each tube when it reaches the set threshold. There is a linear relationship between the Ct value and the logarithmic value of the number of HPV viral copies in the sample. The larger the initial viral copies, the smaller the Ct value [18]. In this study, the Cobas HPV-Ct value, HPV genotyping, cytology results, and the histopathological diagnosis in the database of CHIMUST (Chinese Multi-center Screening Trial) were analyzed. The relationship between the viral load, as reflected by the HPV-Ct value, and pre-invasive cervical lesions was explored. The effectiveness of incorporating viral load (HPV-Ct value) in secondary screening algorithms was also evaluated.

## 1 Materials and methods

### 1.1 Study subjects

**Fig 1:**
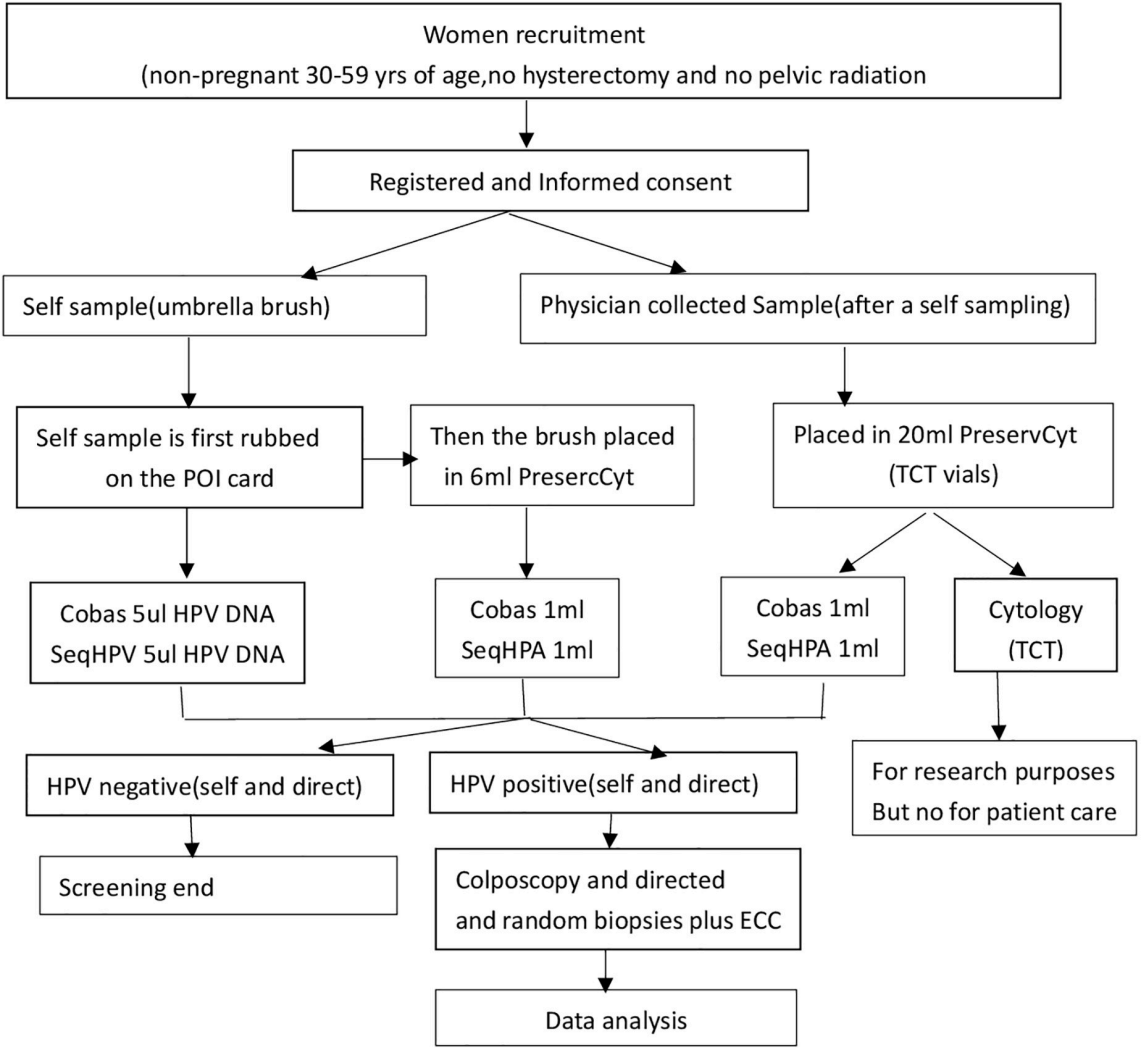
CHIMUST protocol.

This study of viral load uses the data from CHIMUST physician samples. CHIMUST is a multi-center, cervical cancer screening study. (Registration number: ChiCTR-EOC-16008456) The protocol of this trial was approved by the ethics committee of Peking University Shenzhen Hospital(IRB:PUSH2016001) and Cleveland Clinic Institutional Review Board (IRB:15–1549). A total of 10,000 non-pregnant sexually active women, ages 30 to 59 years were recruited. Study entry required no prior pelvic radiation, no hysterectomy, and no cervical cancer screening in the past 3 years. Women were recruited from 6 provinces and cities in China. All participants signed an informed consent document before enrollment. All participants provided a self-collected vaginal sample and a physician-collected endocervical sample. The sample on the self-sampling brush was transferred to the POI card [19] (POI card sample), and then the self-sampling brush was placed in a small bottle containing 6mL of PreservCyt^®^ solution (Hologic, Marlborough, Mass. USA). The physician-sampling was performed following self-sampling. The physician placed a vaginal speculum to expose the cervix, then obtained a cervical exfoliated cell sample at the squamocolumnar junction of the cervix with a sampling brush and then placed the brush into a 20mL PreservCyt^®^ solution (DOC sample) for testing. All samples were tested with the PCR-based high-risk HPV assays: Cobas and SeqHPV (BGI, Shenzhen, China). The physician-collected samples were also tested by Cytology using the Hologic I2 imager system (computer assisted cytology). Cytology was used as a secondary screening to triage HPV positive women. (see Fig 1 for the process).

### 1.2 Study methods

#### 1.2.1 Cobas testing

HR-HPV testing was performed on Cobas 4800. The instrument consists of Cobas Z480 amplification analyzer and Cobas X480 automatic nucleic acid extractor (Roche Diagnostics USA). It can be used for full-automatic sample preparation and real-time PCR amplification to detect a total of 14 HR-HPV subtypes, including HPV 16, 18, 31, 33, 35, 39, 45, 51, 52, 56, 58, 59, 66 and 68, and provides the results of HPV 16 and HPV 18, and the pooled results of the other 12 subtypes in the assay. It can also provide the cycle threshold (Ct value) of the virus copy number,that can reflect the viral load. All procedures were carried out in strict accordance with the working manual of the testing technology and the guidelines for the companion kit.

#### 1.2.2 Pathological diagnosis of colposcopic biopsy

Patients testing HPV positive for any HPV sample (self or direct on either assay) were called back for colposcopy, and evaluated using POI biopsy protocol of directed and random biopsies plus endocervical curettage (ECC) [20]. Both colposcopists performed colposcopy and pathologists made histological diagnosis blingding to the cytology. Pathological diagnosis included negative (for intraepithelial lesion/malignancy), CIN1, CIN2, CIN3, microinvasive cancer, and invasive cancer. The highest pathological grade was taken as the final pathological diagnosis.

### 1.3 Statistical analysis

SPSS v.22.0 software(IBM, Armonk, NY, USA) was used for all data analysis in this study with the significance level α = 0.05. Quantitative data were described by mean ± standard deviation and compared by one-way ANOVA. Pearson correlation analysis was used to analyze the correlation of quantitative data. A logistic regression model was used to analyze the relationship between covariates and the probability of CIN2+ in HR-HPV positive women; the OR was adjusted for covariates. McNemar was used to analyze the differences between the sensitivity and specificity for CIN2+ detection and CIN3+ detection in the paired sample groups, and when *P*<0.05, the differences were interpreted as statistically significant.

### 1.4 Data availability

All relevant data are within the manuscript. Authors did not access to information that could identify individual participants during or after data collection.

## 2 Results

### 2.1 Overview of data

From August 2016 to January 2018, 10885 eligible women were screened with an average age of 43.9 years. 80.6% (1650/2046) of the participants testing positive on one or more tests returned for colposcopy. Except for 396 cases of colposcopy without returning, 90 cases of any HPV assay failed,10399 had complete data and are therefore included in this analysis. The pathological diagnosis showed: normal cases (1,331), CIN1 (177), CIN2 (88), CIN3 (49) and cervical cancer (5). The enrolled subjects were divided into different arms according to the pathological results: 1,508 cases of ≤CIN1 (normal and CIN1), 142 cases of CIN2+ (CIN2, CIN3 and cancer), and 54 cases of CIN3+ (CIN3 and cancer).

Cobas-HPV positive rate was 10.78% (1121/10399); The positive rates of HPV16, HPV18 and non-16/18 HPV were 1.96% (204/10399), 0.72% (75/10399) and 8.94% (930/10399) respectively; 136, 52 and 850 patients were only infected with HPV16, HPV18 and the other 12 types respectively.

### 2.2. Ct values of HPV subtype infections in cervical lesions of all grades

There were significant differences in the Ct values of HPV16 and 12 other HR-HPV subtypes in cervical lesions of different grades (*P*<0.001). After pairwise comparisons, it was found the Ct values of HPV16 and 12 other HR-HPV subtypes in ≤CIN1 lesions were higher than those in CIN2 and CIN3+ lesions, but there was no statistically significant difference between CIN2 and CIN3+ lesions (*P*>0.05). The Ct values of HPV16 and 12 other HR-HPV subtypes gradually decreased with the increase in the grade of cervical lesions (HPV16: *r* = -0.429, *P*<0.001; 12 other HR-HPV subtypes: *r* = -0.099, *P*<0.01). HPV18 infection caused only 7 cases of CIN2+ lesions. There was no statistically significant difference in the HPV18-Ct value between all grades of cervical lesions (*P*>0.05) and there was no correlation between the HPV18-Ct value and the grade of cervical lesions (*r* = 0.089, *P* = 0.446). See Table 1.

**Table 1:** Ct Values of HR-HPV subtype infections in different grade of cervical lesions (mean ± standard deviation).

Pathological grade	HPV16*		HPV18		Non-16/18HPV*	
	Number of cases	Ct value	Number of cases	Ct value	Number of cases	Ct value
≤CIN1	139	31.5±4.46	68	31.4±4.19	839	31.4±5.09
CIN2	29	28.2±3.90	6	35.0±4.27	62	29.4±4.68
CIN3+	36	26.8±2.75	1	26.5	29	29.7±5.65

*P<0.05

### 2.3 Risk of CIN2+ in Ct values of HPV16 and 12 other HR-HPV subtypes

The risk of CIN2+ was not predicted using a threshold with different HPV18-Ct values (limited by only 7 cases). With the quartile of the Ct values of HPV16 and 12 other HPV-Ct subtypes as the threshold, the HPV-Ct values were divided into three levels, namely, Q1: ≤25% percentile, Q2: ≤50% percentile, Q3: ≤75% percentile. See Table 2 for the detailed quartile critical Ct values of HPV16 and 12 other HPV subtypes.

**Table 2:** Quantiles of the Ct values of HPV16 and 12 other HPV subtypes.

	25% percentile	50% percentile	75% percentile
HPV16	27	30	33
12 other HPV subtypes	27	31	35

The risk of CIN2+ in HPV16-positive women increased significantly with the decrease of HPV-Ct value (i.e. the increase of viral load) as compared with that in HPV16-negative women. The risk of CIN2+ in women who were positive for 12 other HPV subtypes also increased with the decrease of HPV-Ct value (i.e. the increase of viral load) as compared with that in women who tested negative for 12 other HPV subtypes. The risk of CIN2+ in women who were positive for 12 other HPV subtypes at all levels of Ct values was lower than that in women who were positive for HPV16. See Table 3.

**Table 3:** Risk of CIN2+ at different levels of Ct values of HPV16 and 12 other HPV subtypes (OR, 95% CI).

	Q1	Q2	Q3
HPV16	171.4(100.4,331.9)	128.9(83.8,209.1)	88.6 (59.3,132.4)
12 other HPV subtypes	30.1(17.2,48.4)	28.5(19.5,42.1)	22.5(16.0,32.8)

### 2.4 Application of HPV subtype and vial load in the triage of HPV-positive women for primary cervical cancer screening

Table 4 presents screening with HPV only, followed by 2 common algorithms to triage HPV positive women with secondary cytology. These data are then compared to 3 algorithms using viral load (HPV-Ct value) without using cytology. The parameters described are the colposcopy referral rate (%), the cytology testing rate (%), and the sensitivity and specificity for CIN2+ and CIN3+. P values compare the viral load algorithms to algorithm #4. Using viral load reflected by Ct values to determine colposcopy referral of the 12 pooled viral types after referral of all 16/18 positives, Ct values of <27, <31, and <35 (algorithm #5, #6, #7) referred a total of 4.40%, 6.59%, and 8.57% respectively. The sensitivities for CIN2+ and CIN3+ of algorithm #5, #6, #7 were 66.9%, 80.3%, 86.6% and 81.5%, 92.6%, 96.3% respectively. The specificities for CIN2+ and CIN3+ of algorithm #5, #6, #7 were 81.5%, 92.6%, 96.3% and 96.0%, 93.9%, 91.9% respectively. Compared with algorithm #4, algorithm#6, #7 had sensitivities with no statistical differences for CIN2+ and CIN3+ (P>0.05), and slightly lower specificities for CIN2+ and CIN3+ (P<0.05). They had higher colposcopy referral rates (P<0.05), but did not require cytology. Algorithm#5 had lower sensitivity and slightly higher specificity for CIN2+ and CIN3+ (P<0.05), with lower colposcopy referral rates (P<0.05). (See Table 4).

**Table 4:** Comparison of different screening strategies.

Screening Algorithms	Colposcopy referral rate %	Cytology testing rate %	CIN2+		CIN3+	
			Sen%	Spe%	Sen%	Spe%
1.CobasHPV+	10.78*	NA	95.1* (135/142)	90.4* (9271/10257)	100 (54/54)	89.7* (9278/10345)
2.HPV16/18+	2.61*	NA	49.3* (70/142)	98.0* (10056/1025)	68.5* (37/54)	97.7* (10111/10345)
3.HPV+ then Cyto≥ASCUS	3.39*	10.8*	73.2* (104/142)	97.6%* (10008/10257)	100 (54/54)	97.1* (10046/10345)
4.HPV16/18+ then Cyto≥ASCUS of 12 other subtypes	4.90	8.2	83.1 (118/142)	96.2 (9865/10257)	100 (54/54)	95.6 (9889/10345)
5.HPV16/18+ then HPV-Ct≤27 of 12 Other subtypes	4.40*#	NA	66.9*# (95/142)	96.5*# (9894/10257)	81.5*# (44/54)	96.0*# (9931/10345)
6.HPV16/18+ then HPV-Ct≤31 of 12 Other subtypes	6.59*	NA	80.3 (114/142)	94.4* (9686/10257)	92.6 (50/54)	93.9* (9710/10345)
7.HPV16/18+ then HPV-Ct≤35 of 12 Other subtypes	8.57*#	NA	86.6# (123/142)	92.5*# (9489/10257)	96.3 (52/54)	91.9*# (9506/10345)

**Abbreviations**: algorithm#1: if CobasHPV positive, refer to colposcopy;

algorithm #2: if HPV16/18 positive, refer to colposcopy

algorithm #3: if HPV positive, underwent cytology, cytology≥ASCUS, refer to colposcopy

algorithm#4: if HPV16/18 positive, refer to colposcopy;12 other HPV subtype positive, underwent cytology, cytology≥ASCUS, refer to colposcopy

algorithm #5: if HPV16/18 positive, refer to colposcopy;12 other HPV subtype positive, HPV-Ct ≤27 of 12 other subtypes refer to colposcopy

algorithm #6: if HPV16/18 positive, refer to colposcopy;12 other HPV subtype positive, HPV-Ct ≤31 of 12 other subtypes refer to colposcopy

algorithm #7: if HPV16/18 positive, refer to colposcopy;12 other HPV subtype positive, HPV-Ct ≤35 of 12 other subtypes refer to colposcopy

*: Compared with algorithm#4, P < 0.05;

#: When algorithm#5 and #7 were compared with algorithm#6, P < 0.05.

## 3 Discussions

Studies on viral load must be based on genotyping to obtain meaningful data concerning the correlation between viral load and cervical pre-cancer and cancer. The relationship between viral load and cervical lesions has been reported to be related to HPV type [21]. In the early stages of this study, our team used Cobas HPV-Ct value as the indicator for viral load to evaluate the relationship between the viral loads of HPV 16, 18 and 12 other HPV subtypes (as a pooled report), and the grade of cervical lesions. These data were obtained in a screening program in Buji, Shenzhen and showed type-specific HPV viral load was closely related to the grade of cervical lesions [22]. Then we further evaluated the relationship between Cobas HPV-Ct value and cervical lesions among the large sample population screened at multiple centers nationwide. The results were consistent with the findings of our earlier study, which further confirmed that the risk of CIN2+ in women who were positive for HPV16 and/or 12 other HR-HPV subtypes increased with the decrease of the corresponding HPV-Ct value (i.e. the increase of viral load). This is consistent with the conclusion of previous reports that the HPV viral load could be used to predict the risk of CIN [16,23]. In view of the high correlation between HPV-Ct values and the grade of cervical lesions, viral load (HPV-Ct value) may be used as a reference index for triaging patients who are HPV positive in primary screening.

In the Provisional Guidelines for Cervical Cancer Screening issued by the Society of Gynecologic Oncology and the American Society for Colposcopy and Cervical Pathology in 2014, sequential screening of combined HPV16/18 and cytology was recommended as a secondary triage plan for women who were HPV positive in primary screening [2]. In this study, we use it as Program #4 which had higher sensitivities for CIN2+ and CIN3+ than the same program used in the ATHENA Study (63.6%, 72.0%) [24]; the reason may be that the Hologic I2 instrument system with computer-aided cytology diagnosis was used, and strict quality control set-up by our senior cytopathologist. The prerequisite for triaging HPV-positive patients by cytology is to have a high quality cytology program which is often unattainable in areas where cytology resources are scarce. In addition, depending how the program is organized, it may require a second visit for cytology, which will further increase the screening costs and the rate for loss to follow-up.

The results of this study have confirmed that the Ct values of the 12-type pool in Cobas are closely related to the grade of cervical lesions. Therefore, we explored different threshold Ct values of the 12-type pool to triage women infected with 12 other types of HPV among the primary screening population who were HPV positive. Algorithm 5 of direct referral for colposcopy for HPV16/18-positive women and then referral for colposcopy for those who were positive for 12-type pool (Ct value≤27), had low sensitivities for CIN2+ and CIN3+, and a missed diagnosis rate of 33% for CIN2+ and nearly 20% for CIN3+, with 10 missed CIN3 cases. Due to the low natural reversal rate of CIN3, missing 33% is not suitable for triaging HPV-positive women. Algorithms 6 and 7 of direct referral for colposcopy for HPV16/18-positive subjects and referral for colposcopy for those from the 12-type pool with HPV-Ct values ≤31/35, had similar sensitivities and slightly lower specificities for CIN2+ and CIN3+ as compared with the guideline (Algorithm 4). Algorithm 6 had a reduced colposcopy referral rate of 6.59% as compared with primary screening (10.78%) while Algorithm 7 had a colposcopy referral rate which is reduced by 2.2% as compared with primary screening and increased as compared with Algorithm 6. In addition, Algorithm 6 had a similar sensitivity for CIN3+ as compared with Algorithm 7. As a result, the Algorithm 6 was considered to be better than #7, although #6 had slightly lower specificities for CIN2+ and CIN3+ as compared with the program recommended by guideline (#4), but it had similar sensitivities. The critical benefit is that the secondary screening can be completed with a single test, without adding cytology. It may reduce the number of return visits and the cost of a second examination depending how the primary sample was managed, and is clearly advantaged in areas lacking cytological resources. Therefore, the sequential screening with combined HPV16/18 and 12 other HPV types with the Ct value ≤31 can be considered as an alternative to cytology-based secondary screening.

This study is the first to use HPV-Ct value to reflect the viral load as a secondary triage for HPV positive women The results may be biased due to the difference in the count of cervical exfoliated cells in the clinical sample. Therefore, in the large sample population screening, we obtained cervical exfoliated cell samples by a standardized sampling procedure of removing cervical secretions and rotating brush 3 times. Although we recognize this method cannot guarantee obtaining the same number of cells.

In conclusion, the secondary screening algorithm of combining HPV16/18 subtype and the viral load of 12 other HPV subtypes (cut-point HPV-Ct≤31) for women who are HPV positive in primary screening, yields the same sensitivity as the currently used sequential secondary screening strategy of HPV16/18 and cytology, and no cytology examination is required. The secondary screening can be completed with a single test. As a result, it is especially suitable for areas lacking cytological resources.

## Supporting information

S1 Checklist (DOCX)

S1 File (RAR)

S2 File (DOCX)

S3 File (PDF)

S4 File (DOCX)

S5 File (DOCX)
