# Supplementary material for: The effectiveness of HPV viral load, reflected by Cobas 4800 HPV-Ct values for the triage of HPV-positive women in primary cervical cancer screening: Direct endocervical samples
Source: PLoS One. 2020 May 7;15(5):e0232107. doi: 10.1371/journal.pone.0232107 (PMC7205204; doi:10.1371/journal.pone.0232107)
Supplement: S2 File — (DOCX) [file pone.0232107.s003.docx]

**Ethics approval form of Peking University Shenzhen Hospital**

Application Department Gynecology and Obstetrics NO： 2016001

| Project type | □research proposal√□Paper Publication□Clinical application of diagnosis and treatment project | | | Name of the journal to be published | | |
| --- | --- | --- | --- | --- | --- | --- |
|  |  |  |  |  | | |
| project title | The Chinese Multi-Center Screening Trial (CHIMUST)  *The Prevention of Cervical Cancer Using Self-collection as the Primary Screen* | | | | | |
| Principal Investigator | Wu Ruifang | specialty | GYN & OBS | | duty | Professor and Chairman |
| Project Abstract | CHIMUST was a nationwide multi-center cervical cancer prevention projects for doctor sampling and self-sampling HPV testing that implemented by Peking University Shenzhen Hospital (PUSH), American International Anti-Cancer Organization (POI), and Cleveland Medical Center as leading units,Peking University People's Hospital, Shanghai Huashan Hospital affiliated to Fudan University, Wuhan University People's Hospital (Hubei Provincial People's Hospital), Hebei Medical University Second Hospital, and Inner Mongolia Autonomous Region People's Hospital as participating units. The objectives of CHIMUST were:  1）To evaluating the sensitivity and specificity for CIN2+ of two assays of the Cobas 4800 HPV and SEQ HPV for testing high-risk HPV in physician-collected samples and vaginal self-samples in screening.  2）To evaluate the effectiveness of different sample collection media, as well further explore cytology,genotyping and viral load as secondary screens. | | | | | |
| The significance of the project | To establish a cervical cancer screening model for self-sampling HPV testing in screening population, which will simplify the screening process, reduce costs, and facilitate women's participation in screening; Because of insufficient specificity of HPV as primary screening, to explore the use of HPV16/18 combined cytology or viral load for secondary strategy can further improve secondary screening, effectively implement early diagnosis and early treatment of cervical cancer, and reduce mortality of cervical cancer. For the first time, the evaluation of the dry brush sample storage method will provide a valuable test basis for further reducing the cost of screening and the popularity of screening in remote areas. | | | | | |
| Possible side effects, hazards and countermeasures of project implementation (including informed consent, protection of privacy, physical damage, distribution of benefits, etc.) | You may feel slightly uncomfortable when taking a doctor sampling. In most cases, you will not feel any discomfort. You may feel slight discomfort during a cervical biopsy. There may be bleeding and infection after the biopsy, but these risks are very low because the biopsy tissue is very small . Once it happens,hemostatic and anti-infective treatment appears. | | | | | |
| Whether the project implementation is ethical | √Respection √benefit (no harm) √Justice | | | | | |
| Possible Ethical Problems and Countermeasures Caused by Project Implementation | The implementation of the project will not cause physical damage and interest distribution. It will strictly abide by the principle of scientific research confidentiality, and will not publish any personal information of patients to protect patient privacy. | | | | | |
| Principal Investigator signment： Wu Ruifang | | | | | | |
| Ethics Committee opinion：  Ethical review results： √agreement □Revision review □disagreemnet  Signed by the chairman of the ethics committee： Wang Tao Aug.15^th^,2016 | | | | | | |
