# Supplementary material for: The effectiveness of HPV viral load, reflected by Cobas 4800 HPV-Ct values for the triage of HPV-positive women in primary cervical cancer screening: Direct endocervical samples
Source: PLoS One. 2020 May 7;15(5):e0232107. doi: 10.1371/journal.pone.0232107 (PMC7205204; doi:10.1371/journal.pone.0232107)
Supplement: S3 File — (PDF) [file pone.0232107.s004.pdf]

Cleveland Clinic Institutional Review Board (IRB)
Federalwide Assurance (FWA 00005367)

Cleveland Clinic

Full AAHRPP Accreditation
Association for the Accreditation of Human Research Protection Programs, Inc.

February 17, 2016

Jerome L. Belinson, M.D.

RE: IRB# 15-1549: The Chinese Multi-Center Screening Trial (CHIMUST) (Preventive Oncology International under a collaboration agreement with Ob/Gyn and Women's Health Institute in cooperation with Peking University Shenzhen Hospital; funding for the 3F project by the Sh)

Dear Dr. Belinson:

Your submission on 2/15/2016 of the Protocol Ver. 9 / 11A dated 2/1/2016, revised Consent dated 2/1/2016, tracked changes, and Chinese Protocol (clean & tracked) was **approved** under expedited review on 2/16/2016.

Protocol changes including a change in specimen collection and other clarifications, as well as consent revisions.

The stamp-approved Informed Consent is available online under the Stamped Documents tab.

Written consent is required to document that each person has been adequately informed about this research and voluntarily agrees to participate prior to any involvement in the research.

Re-consenting is not applicable. New subjects only.

If you have any questions or concerns, you may contact the IRB Office at 216-444-2924 or email IRB@ccf.org.

The study expiration date of 1/7/2017 remains unchanged.

Sincerely,

Bridget Howard

Bridget Howard, J.D.
Executive Director, IRB and Human Research Protections

BH:ll/so

Expiration Date: 1/7/2017

This letter is available online under the Correspondence tab
